# Malaysian pharmacy students’ perspectives on the virtual objective structured clinical examination during the coronavirus disease 2019 pandemic

**DOI:** 10.3352/jeehp.2021.18.6

**Published:** 2021-04-12

**Authors:** Mohamed Hassan Elnaem, Muhammad Eid Akkawi, Nor Ilyani Mohamed Nazar, Norny Syafinaz Ab Rahman, Mohamad Haniki Nik Mohamed

**Affiliations:** Department of Pharmacy Practice, Faculty of Pharmacy, International Islamic University Malaysia, Kuantan, Malaysia; Hallym University, Korea

**Keywords:** COVID-19, Educational measurement, Malaysia, Pharmacy students, Physical examination

## Abstract

**Purpose:**

This study investigated pharmacy students’ perceptions of various aspects of virtual objective structured clinical examinations (vOSCEs) conducted during the coronavirus disease 2019 pandemic in Malaysia.

**Methods:**

This cross-sectional study involved third- and fourth-year pharmacy students at the International Islamic University Malaysia. A validated self-administered questionnaire was distributed to students who had taken a vOSCE a week before.

**Results:**

Out of the 253 students who were approached, 231 (91.3%) completed the questionnaire. More than 75% of the participants agreed that the instructions and preparations were clear and helpful in familiarizing them with the vOSCE flow. It was found that 53.2% of the respondents were satisfied with the flow and conduct of the vOSCE. However, only approximately one-third of the respondents believed that the tasks provided in the vOSCE were more convenient, less stressful, and easier to perform than those in the conventional OSCE. Furthermore, 49.7% of the students favored not having a vOSCE in the future when conducting a conventional OSCE becomes feasible again. Internet connection was reported as a problem hindering the performance of the vOSCE by 51.9% of the participants. Students who were interested in clinical pharmacy courses were more satisfied than other students with the preparation and operation of the vOSCE, the faculty support, and the allocated time.

**Conclusion:**

Students were satisfied with the organization and operation of the vOSCE. However, they still preferred the conventional OSCE over the vOSCE. These findings might indicate a further need to expose students to telehealthcare models.

## Introduction

### Background/rationale

With the global transformation of pharmacy practice to be more patient-focused, there has been a noticeable trend towards increasing the clinical components in pharmacy education programs. Consequently, the objective structured clinical examination (OSCE) has become a key assessment method used by pharmacy schools worldwide. The OSCE has also been adopted by several professional associations of pharmacists for certification and licensing purposes, as it enables the assessment of pharmacists and future pharmacists across a wide range of real-life clinical scenarios [1]. In designing OSCE cases, there should be a simulated interaction between the student and healthcare provider or patients to enhance students’ communication skills and practical application of their knowledge [1]. In the typical scenario of conducting an OSCE, a series of interactive face-to-face sessions are designed to simulate real situations in the practice settings [1,2]. However, the coronavirus disease 2019 (COVID-19) pandemic has imposed several restrictions on face-to-face interactions, creating challenges for pharmacy schools to pursue their planned conventional OSCEs. The virtual OSCE (vOSCE), as a proposed innovative method in experiential learning and assessment, then emerged as a way to proceed with the OSCE tasks without involving any face-to-face interactions with students. Careful planning for the online set-up and flow, training for students and assessors beforehand, clear instructions for the students, and simultaneous technical support are essential factors for a successful vOSCE. With slight modifications and creativity, it is possible to assess online almost all skills usually included in a face-to-face OSCE [3]. Trials also showed that good planning and training could overcome common hurdles to successful OSCEs, such as a large number of students, the use of simulated patients, and the operation of multiple concurrent stations [4].

With the unexpected COVID-19 pandemic, many emergency learning and evaluation initiatives seem to be continuing for a more extended period than previously planned. The implementation of online assessments during the pandemic has received considerable interest, and students’ feedback has been a particular focus of attention as part of lessons learned for potential improvements [5]. To further improve learning and evaluation methods, it is crucial to receive feedback from students on newly implemented online assessments.

### Objectives

This study investigated Malaysian pharmacy students’ perceptions and views regarding the vOSCE conducted to assess their skills and competencies during the COVID-19 pandemic, with a specific focus on the preparation, organization, conduction, and advantages/disadvantages of the vOSCE compared with the conventional face-to-face OSCE.

## Methods

### Ethics statement

The study protocol obtained an ethical approval recommendation from the faculty of Pharmacy Ethics Committee (KEC) (review no., 4/2019) and obtained ethical approval from the International Islamic University Malaysia (IIUM) Research Ethics Committee (IREC 2020-074). Informed consent was obtained from participants.

### Study design

A descriptive cross-sectional study was conducted to explore the views and perceptions of pharmacy students in Malaysia.

### Participants

The target participants of the study were penultimate and final-year students of the Faculty of Pharmacy, IIUM in Malaysia. All 253 students (140 year 3 and 113 year 4) who took part in the vOSCE were invited to participate in the study. Participation was voluntary, and no payment was given.

### Setting

The vOSCE was conducted in response to changes in the assessment methods used at IIUM during the COVID-19 pandemic. A validated questionnaire was distributed to the students 1 week after they took the vOSCE. All potential study participants were provided with a link to the online questionnaire (using a Google form), with at least 3 reminders to enhance the response rate.

In total, 3 vOSCEs were conducted in July 2020, covering different modules in clinical, hospital, and community pharmacy services courses. A similar approach was followed for all vOSCEs; each group of students was assigned to a faculty member as an examiner, and a trial-run session was arranged a day before the vOSCE. The examiners and students had the option to choose either Google Meet or Zoom as the platform of choice to conduct the assessment. The cases for assessment were shared with the students by the examiners using the screen-sharing option.

### Development, validation, and reliability of the measurement tool

A self-administered 22-item structured English questionnaire was developed, as no similar tool to achieve the research objectives was found in the literature. The content validity of the developed tool was evaluated through an examination by 5 senior academic staff with experience matching the research topic. Accordingly, the content validity index for each item was calculated, and input from the validators was considered to make the necessary amendments. Based on their input, 4 items were deleted from the proposed questionnaire, which consisted of 26 items. The internal consistency and reliability of the original questionnaire were tested and reported previously, with a Cronbach’s α value of 0.87 [6].

The study instrument was divided into 3 sections. Section A comprised 5 items eliciting general information about the study participants. Section B contained 17 items used to assess students’ views and perceptions regarding the vOSCE. The items in section B were designed to encompass students’ perceptions on 5 domains of the vOSCE: the efforts made by faculty to prepare the students; the operation of the vOSCE; technical issues and faculty support in the operation of the vOSCE; normalization of the vOSCE in the future; and a comparison between conventional OSCE and vOSCE. Section C consisted of an open-ended space to provide relevant comments about the overall vOSCE experience. The items in section A were designed mainly as discrete items, whereas the items in section B were answered on a 5-point Likert scale, with a score of 1 denoting “strongly disagree” and a score of 5 indicating “strongly agree.”

### Study size

A sample size of 153 students was calculated to represent the finite population of 253 pharmacy students with a response distribution of 50%, a margin of error of 5%, and a level of confidence of 95%. This sample size achieved a statistical power of 96% and 99% for gender and year of study, respectively. The sample size was calculated using the Raosoft online sample size calculator (Raosoft Inc., Seattle, WA, USA).

### Statistical methods

The data were analyzed using IBM SPSS software ver. 23.0 (IBM Corp., Armonk, NY, USA). Descriptive data were expressed as numbers and percentages. The chi-square test was utilized to compare students’ responses based on their characteristics. The level of significance was set at P<0.05.

## Results

Out of 253 students approached, 231 answered the questionnaire, corresponding to a response rate of 91.3%. The respondents’ characteristics are summarized in Table 1. Raw response data are available in Dataset 1.

The statements of the questionnaire were categorized into 5 different domains, as listed in Table 2. Regarding the effort made by faculty to prepare the students, more than 75% of the participants agreed that the instructions, briefing, and trial run sessions conducted before the vOSCE were clear and helpful in familiarizing them with the vOSCE flow. Furthermore, 60.6% of the respondents agreed that the examiner’s interaction during the trial-run reduced their level of stress. Pertaining to the operation of the vOSCE, 53.2% of the respondents were satisfied with the flow and conduct of the vOSCE. However, only 32.5% of the respondents were satisfied with the allocated time for each task given.

Approximately one-third of the respondents believed that the tasks provided in the vOSCE were easier to perform, more convenient, and less stressful than those in the conventional OSCE. A similar proportion agreed that the vOSCE allowed them to demonstrate their knowledge and skills to the same extent as the conventional OSCE. However, 53.7% of the respondents indicated that they would choose to have a face-to-face session over a video conferencing platform with the examiners, while 34.6% believed that they would achieve better in a conventional OSCE than in the vOSCE. In terms of technical issues, 51.9% of the participants indicated that the internet connection was a problem affecting their performance in the vOSCE. Nevertheless, a majority of the students (68.1%) believed that the faculty had provided ample support when they encountered technical difficulties.

For the last domain, on normalizing the vOSCE in the future, only 32 students (13.9%) favored the possibility of conducting future OSCEs virtually, although 63 students (27.3%) agreed that the vOSCE enhanced their confidence in dealing with patients or other healthcare professionals. The overall responses of the respondents are presented in Table 2.

The analysis showed significant differences in the responses to some questions based on gender or year of study. For instance, 58.6% of year 3 students found that the flow and conduct of the virtual OSCE were smooth and appropriate compared to 46.6% of year 4 students (P=0.0058). Furthermore, 87% of the female students agreed that the OSCE protocol was clear, compared to 76% of male students (P=0.001). Table 3 presents the statements for which a significant difference in the response was found. Students’ interest in clinical pharmacy courses (CPCs) also affected their feedback on the vOSCE. Overall, students interested in CPCs were more satisfied than other students in terms of vOSCE preparation, conduct, faculty support, and the allocated time (Table 3).

Ninety students (39%) answered the open-ended question eliciting feedback on the vOSCE. The comments were classified based on their relevance to the questionnaire sections. The majority of the commenters (79%) complained that the case file was not downloadable, so it would have been more convenient to go through the file during the allocated time. Therefore, the students preferred using an online platform that would enable them to control the case file remotely. All other comments highlighted the same aspects that were covered by the questionnaire, such as time allocation, internet connection, and other technical issues.

## Discussion

### Key results

The findings highlighted the students’ overall perceptions concerning the flow of the vOSCE, including the arrangements made by faculty, their comparative views of the vOSCE versus the conventional OSCE, the technical issues associated with the virtual setting, and the adoption of the vOSCE in future assessments. More than half of the respondents were satisfied with the flow and conduct of the vOSCE. However, only one-third of them said that the tasks provided in the vOSCE were more convenient, less stressful, and easier to perform than those in the conventional OSCE. Approximately half of the students favored not using the vOSCE in the future if a conventional OSCE is available.

### Interpretation

According to recently published recommendations for conducting online OSCEs, detailed planning and proper use of workforce and resources are essential for preparing a smooth and convenient assessment experience [7]. In our study, the majority of students had positive perceptions concerning the faculty initiatives in planning for the vOSCE. Our study participants commended the beneficial role of a written protocol and compulsory trial-run sessions involving all students and their proposed examiners. From students’ perspectives, the examiner’s prior communication in a mock session reduced the students’ stress level before the assessment. Moreover, more than half of the responses agreed that the overall vOSCE process flow was appropriate. However, about 40% of the participants disagreed that the time allocated for their tasks was sufficient. It seems that the vOSCE could not overcome the long-term concern of time adequacy, which has been highlighted frequently regarding conventional OSCE assessments [8]. It would be recommended to ensure time adequacy by reviewing the assessment tasks and allocated time, including the additional time needed as part of the online technical requirements.

Concerning comparative perceptions on the conventional OSCE and vOSCE, the study participants highlighted several differences. More than half of the respondents viewed the direct face-to-face interaction with the examiner in a conventional OSCE as more convenient than using an online platform in the vOSCE. Unlike our study participants, dentistry and medical students from North America agreed that their experience of vOSCE was comparable and as successful as the conventional OSCE [5,9]. This may be attributed to the low exposure of our students to online assessment activities before the pandemic. Consistently, half of the responses disagreed with maintaining the vOSCE in the post-pandemic era when the conventional OSCE could be conducted. As reported previously in the local setting, our findings indicate that the students viewed the simulation of real clinical practice involving direct interactions between healthcare providers and patients as preferable to an online assessment model [10]. In addition, only about one-third of the students agreed that the vOSCE allowed them to demonstrate their knowledge and skills to the same degree as the conventional OSCE. This result disagrees with a study of dentistry and nursing students in the United States, where the majority of the participants felt they were able to fully showcase their knowledge through the vOSCE [5]. This might also indicate the further need to expose our students to telehealthcare models in order to familiarize them with ways of demonstrating their clinical skills and providing healthcare services remotely.

Concerning the stress associated with the OSCE, it has been frequently highlighted that OSCEs are often associated with remarkably higher stress levels than are associated with other assessment types [11]. The OSCE tends to be perceived as a more difficult type of examination than others [12]. Our findings showed that about 44% and 41% of the respondents disagreed that the vOSCE was less stressful and easier, respectively, than the conventional OSCE. This finding might imply that the source of stress and difficulty levels perceived for the OSCE may be more closely related to the nature of the given tasks, rather than the medium of communication.

As expected, more than half of the responses referred to the quality of the internet connection, which impacted their vOSCE experience. This finding is not an isolated case, especially in Southeast Asia, where problems such as bandwidth issues, connectivity, financial limitations, the digital divide, learners’ engagement, technological adaptation, and capabilities exist in every level of online and remote learning [13]. Although connection problems were common in online assessments [5], most respondents were satisfied with the planned arrangements to overcome such barriers by adjusting the time or changing the online platform. In the era of online education, which started in earnest with the pandemic, establishing an alternative arrangement during online assessments is essential. It would be helpful to convey to the students that their faculty members are considering the potential limitations that they may face, and are ready to support students as needed.

### Comparison with previous studies

Previous research has not provided conclusive data on the impact of students’ characteristics, such as gender, on the OSCE assessment [6]. Our findings showed a few differences among the study participants based on gender, year of study, and their interest in CPCs. Female participants tended to have better perceptions regarding the clarity of the instructions before the assessment. Thus, they showed more appreciation for the examiners’ support throughout the vOSCE experience than male participants. Year 3 students were more likely to agree than year 4 students that the overall flow of the vOSCE was smooth. This finding could be explained by the fact that the vOSCE for year 3 was designed as 1 task; meanwhile, the vOSCE for year 4 students was designed as 2 tasks to be completed consecutively. This may imply the need to consider refining the number of tasks offered in the vOSCE. Finally, students who expressed an interest in CPCs tended to have better perceptions in several items related to preparation, operation, and faculty support throughout the vOSCE experience. These findings highlight that students’ baseline clinical interest might directly impact their level of satisfaction with clinical learning activities and assessments such as the vOSCE.

### Limitations

The work was conducted at a single public pharmacy school in Malaysia; therefore, it may not be generalizable to vOSCEs at other pharmacy schools in Malaysia or beyond. In addition, there was no further investigation into the possible impact of students’ grades on their satisfaction with the vOSCE.

### Conclusion

This study provides a basis for the better implementation of vOSCEs in the future. Students were satisfied with the overall organization and operation of the vOSCE; however, they still preferred conventional over vOSCEs, indicating a potential need for further exposure to telehealthcare models. Stress and time limitations did not change significantly with the vOSCE compared to conventional OSCE. Students’ self-interest in clinical subjects seemed to have affected their perceptions and level of satisfaction with the experiential learning assessments.

## Figures and Tables

**Table 1.** Characteristics of the participants (n=231)

Characteristic	No. (%)
Gender	
Male	54 (23.4)
Female	177 (76.6)
Year of study	
Year 3	128 (55.4)
Year 4	103 (44.6)
Interest in clinical pharmacy courses	
Yes	180 (77.9)
No/neutral	51 (22.1)
Interest in clinical pharmacy as a career pathway after graduation	
Yes	156 (67.5)
No/neutral	75 (32.5)

**Table 2.** Responses of the participants (n=231)

Statement	Response no. (%)		
	Strongly agree/agree	Neutral	Disagree/strongly disagree
I. Perceptions of faculty effort in preparing the students			
1. The instructions of the virtual OSCE (protocol) were clear.	195 (84.4)	29 (12.6)	7 (3.0)
2. The briefing regarding the overall process of the virtual OSCE before the exam was clear and helpful.	201 (87.0)	22 (9.5)	8 (3.5)
3. The trial-run session with the examiner helped familiarize me with the flow of virtual OSCE.	184 (79.7)	35 (15.2)	12 (5.1)
4. The prior communication with the examiner during the trial-run session helped to reduce my level of stress.	140 (60.6)	66 (28.6)	25 (10.8)
II. Perceptions of the operation of the virtual OSCE			
5. The time provided to complete each task of the virtual OSCE was adequate.	75 (32.5)	66 (28.6)	90 (38.9)
6. Overall, the flow and conduct of the virtual OSCE were smooth and appropriate.	123 (53.2)	66 (28.6)	42 (18.2)
III. Comparison between the conventional OSCE and the virtual OSCE			
7. The virtual OSCE was more convenient to me than the conventional one, as I did not have to move from one room to another.	79 (34.2)	44 (19.0)	108 (46.8)
8. The virtual OSCE was less stressful as compared to the conventional OSCE.	85 (36.8)	44 (19.0)	102 (44.2)
9. I feel that it would be more convenient to interact face to face with the examiners rather than a video call.	124 (53.7)	80 (34.6)	27 (11.7)
10. I feel that the virtual OSCE was as helpful as the conventional OSCE to gain clinical pharmacy-related skills.	88 (38.1)	78 (33.8)	65 (28.1)
11. The virtual OSCE allowed me to demonstrate my knowledge and skills to the same extent as I do in the conventional OSCE.	84 (36.4)	70 (30.3)	77 (33.3)
12. It was easier to perform the tasks needed in the virtual OSCE than in the conventional OSCE.	80 (34.7)	56 (24.2)	95 (41.1)
13. I believe that my achievement would be somewhat similar if the OSCE was conducted through face-to-face interactions with the examiner.	69 (29.9)	82 (35.5)	80 (34.6)
IV. Technical issues and faculty support in the operation of the virtual OSCE			
14. The quality of my internet connection and speed significantly affected my virtual OSCE experience.	120 (51.9)	54 (23.4)	57 (24.7)
15. I believe the examiner provided support (e.g., giving extra time or providing an alternative platform) if any technical problems occurred during the virtual OSCE session.	157 (68.1)	56 (24.2)	18 (7.7)
V. Perception on normalizing the virtual OSCE in the future			
16. I prefer that all future OSCEs be conducted virtually.	32 (13.9)	84 (36.4)	115 (49.7)
17. I feel that the virtual OSCE has helped give me more confidence to deal with patients/healthcare professionals in the future.	63 (27.3)	90 (39.0)	78 (33.8)

OSCE, objective structured clinical examination.

**Table 3.** Significant differences in responses based on respondents’ characteristics (n=231)

Statement	Year of study			Gender			Interest in CPCs		
	Year 3	Year 4	P-value	Male	Female	P-value	Yes	No	P-value
1. The instructions of the virtual OSCE (protocol) were clear.	110 (85.9)	85 (82.5)	0.346	41 (75.9)	154 (87.0)	0.001	153 (85.0)	42 (82.0)	0.406
2. The briefing regarding the overall process of the virtual OSCE before the exam was clear and helpful.	112 (87.5)	89 (88.4)	0.344	45 (83.3)	156 (77.6)	0.149	162 (90.0)	39 (76.5)	0.006
4. The prior communication with the examiner during the trial-run session helped to reduce my level of stress.	71 (55.5)	69 (67.0)	0.111	31 (57.4)	109 (61.6)	0.556	118 (65.6)	22 (43.1)	0.025
5. The time provided to complete each task of the virtual OSCE was adequate.	42 (32.8)	33 (32.0)	0.972	18 (33.3)	57 (32.2)	0.884	67 (37.0)	8 (15.7)	0.025
6. Overall, the flow and conduct of the virtual OSCE were smooth and appropriate.	75 (58.6)	48 (46.6)	0.005	33 (61.1)	90 (50.8)	0.174	102 (56.0)	21 (42.0)	0.145
8. The virtual OSCE was less stressful as compared to the conventional OSCE.	46 (35.9)	39 (37.9)	0.677	21 (33.3)	64 (34.5)	0.791	74 (41.1)	11 (21.6)	0.003
16. I prefer that all future OSCEs be conducted virtually.	64 (61.7)	56 (75.7)	0.003	32 (63.0)	88 (69.5)	0.20	97 (71.1)	23 (54.9)	0.003

Values are presented as number (% of students who agreed with the statements). CPC, clinical pharmacy course; OSCE, objective structured clinical examination.

## Footnotes

**Data availability** Data files are available from Harvard Dataverse: https://doi.org/10.7910/DVN/AWC1IP Dataset 1. Raw response data of the questionnaire on the virtual objective structured clinical examination from pharmacy students at International Islamic University Malaysia.

## Supplementary materials

Supplementary files are available from Harvard Dataverse: https://doi.org/10.7910/DVN/AWC1IP

Supplement 1. Audio recording of the abstract.
